# The analysis of association between single features of small vessel disease and stroke outcome shows the independent impact of the number of microbleeds and presence of lacunes

**DOI:** 10.1038/s41598-024-53500-7

**Published:** 2024-02-10

**Authors:** Arsany Hakim, Laura Gallucci, Christoph Sperber, Beata Rezny-Kasprzak, Eugen Jäger, Thomas Meinel, David Seiffge, Martina Goeldlin, Franziska Westphalen, Urs Fischer, Roland Wiest, Marcel Arnold, Roza Umarova

**Affiliations:** 1 University Institute of Diagnostic and Interventional Neuroradiology, Inselspital, University Hospital Bern, University of Bern, Bern, Switzerland; 2 Department of Neurology, Inselspital, University Hospital Bern, University of Bern, 3010 Bern, Switzerland; 3 Department of Neurology, University Hospital Basel, University of Basel, Basel, Switzerland

**Keywords:** Neuroscience, Neurology

## Abstract

The impact of small vessel disease (SVD) on stroke outcome was investigated either separately for its single features in isolation or for SVD sum score measuring a qualitative (binary) assessment of SVD-lesions. We aimed to investigate which SVD feature *independently* impacts the most on stroke outcome and to compare the continuous versus binary SVD assessment that reflects pronouncement and presence correspondingly. Patients with a first-ever anterior circulation ischemic stroke were retrospectively investigated. We performed an ordered logistic regression analysis to predict stroke outcome (mRS 3 months, 0–6) using age, stroke severity, and pre-stroke disability as baseline input variables and adding SVD-features (lacunes, microbleeds, enlarged perivascular spaces, white matter hyperintensities) assessed either *continuously* (model 1) or *binary* (model 2). The data of 873 patients (age 67.9 ± 15.4, NIHSS 24 h 4.1 ± 4.8) was analyzed. In model 1 with continuous SVD-features, the number of microbleeds was the only independent predictor of stroke outcome in addition to clinical parameters (OR 1.21; 95% CI 1.07–1.37). In model 2 with the binary SVD assessment, only the presence of lacunes independently improved the prediction of stroke outcome (OR 1.48, 1.1–1.99). In a post hoc analysis, both the continuous number of microbleeds and the presence of lacunes were independent significant predictors. Thus, the number of microbleeds evaluated continuously and the presence of lacunes are associated with stroke outcome independent from age, stroke severity, pre-stroke disability and other SVD-features. Whereas the presence of lacunes is adequately represented in SVD sum score, the microbleeds assessment might require another cutoff and/or gradual scoring, when prediction of stroke outcome is needed.

## Introduction

Small vessel disease (SVD) is considered to be a common cause of stroke and vascular dementia^1^. It manifests with various lesions, visible in imaging as small recent subcortical infarctions, lacunes, white matter hyperintensities (WMH), microbleeds, enlarged perivascular spaces (EPVS), and brain atrophy^1^. Among patients with similar degrees of SVD on brain imaging, clinical symptoms are often highly inconsistent in nature and clinical severity^2^. The overall burden of SVD lesions was shown to be associated with functional disability and cognitive performance^3^, and a higher risk of stroke and death^4,5^ even in young adults^6^.

There are two common forms of sporadic SVD: (1) hypertensive-related (arteriosclerotic/ cardiovascular risk factor-mediated) and (2) cerebral amyloid angiopathy^7^. To reflect the overall burden of the former form, the SVD score was introduced, which captures the presence of SVD features and their pronouncement in a binary way based on the given cut-off for major types of SVD lesions: lacunes, microbleeds, WMH, and EPVS^8,9^. The SVD score was demonstrated to be associated with global cognitive and functional impairment in stroke patients^10^ and poorer stroke outcome^11^. There is evidence for an impact on stroke outcome for each major type of SVD feature but EPVS^12^. Thus, the presence and severity of WMH^13^, multiple lacunes^14^ and microbleeds^15^ were shown to worsen stroke outcome. However, these studies assessed the impact of single SVD features without accounting for other ones. Taking into account a typical progression of pathophysiological developments of SVD^1,16^, one can expect that the pronouncement of different SVD features might correlate with each other and thus their *independent* impact on stroke outcome is still to be defined. In addition, whereas mainly simple binary measures for SVD are used, a *continuous* assessment of SVD features could potentially improve the prediction of stroke outcome, as it better reflects the severity of SVD pathology. Many existing studies on the impact of SVD also did not consider the pre-stroke level of disability in their prediction models.

Considering the gaps of knowledge, we aimed (i) to investigate what SVD feature impacts mostly stroke outcome *independently* from other SVD features and beyond clinical baseline characteristics; (ii) to compare continuous versus binary SVD assessment that reflect correspondingly the pronouncement and the presence of SVD specific features.

## Methods

### Patients

In this retrospective single-center cohort study, we included patients from the Bernese stroke register, admitted to the Bern stroke center for treatment of acute ischemic stroke between January 2015 and October 2020. Inclusion criteria were: (i) age ≥ 18 years old; (ii) the first-ever ischemic anterior circulation stroke to avoid heterogeneity introduced by distinct functional recovery trajectories in posterior circulation stroke; (iii) available MRI examination, which has been acquired routinely either at admission or 24 h post-stroke. According to the clinical guidelines, all patients admitted to our stroke center should be examined by MRI at admission or 24 h post-stroke. However, CT scans were acquired instead if the patient’s general condition was instable (e.g. low level of consciousness, instability of blood pressure), contraindications for MRI existed (e.g. due to the pacemaker), and, more rarely, when MR-scanner capacity was limited. Patients who only received CT were excluded from the study, as CT does not allow the evaluation of all SVD features. Exclusion criteria were: (i) new stroke within the 3 months post-stroke, complications, death or other events within 3 months post-stroke that hamper the interpretation of functional outcome 3 months post-stroke; (ii) absent general consent for the data usage and/or missing clinical stroke scores; (iii) additional stroke in the posterior circulation. All patients underwent standardized treatment according to the Bernese Stroke guidelines, as well as standardized rehabilitation after discharge from the stroke unit. The study was approved by the regional ethics committee (Kantonale Ethikkommission Bern). Written informed consent was obtained from all participants. All methods were carried out in accordance with relevant guidelines and regulations.

During the standardized treatment, all patients were assessed by a certified neurologist. The clinical examination included NIH Stroke Scale (NIHSS) at admission and 24 h after stroke onset; the pre-stroke disability and functional stroke outcome 3 months post-stroke were assessed using the modified Rankin Scale (mRS). The cardiovascular risk factors were documented during hospitalization.

### MRI-Imaging and assessment of small vessel disease neuroimaging markers

We analyzed clinical MRI data, which have been acquired routinely either at admission or 24 h post-stroke. MRI examinations were performed on either a 1.5 T or 3 T scanner (Magnetom Avanto, Aera, Skyra, Verio, Vida, Siemens Medical Solutions, Erlangen, Germany). The following MRI sequences from the emergency stroke protocol were used for the analysis: axial diffusion-weighted imaging (DWI) with apparent diffusion coefficient (ADC) (4–5 mm slice thickness), fluid-attenuated inversion recovery (FLAIR, 4–5 mm slice thickness), susceptibility-weighted imaging (SWI, 1.5 mm slice thickness), and axial contrast-enhanced T1-weighted image (4–5 mm slice thickness). Only MRI data that included all necessary sequences—DWI, FLAIR, SWI, T1—were considered for the analysis. Patients with pronounced artifacts in the MRI images were excluded from the analysis.

The four major imaging features of SVD composing SVD score were rated: WMH pronouncement, the total number of lacunes, the total number of microbleeds, and the number of EPVS. We did not evaluate the SVD features of cerebral amyloid angiopathy such as cortical superficial siderosis as our focus was hypertensive/age-related SVD, due to the low probability of cerebral amyloid angiopathy in ischemic stroke populations and potential confounders such as previous head trauma etc. Lacunes were defined as cerebrospinal fluid isointense ovoid or round cavity ranging from 3 to 20 mm in diameter with surrounding gliosis seen as hyperintensity on the FLAIR image^9,12^. Microbleeds were defined as a small homogenous hypointense signal on the SWI sequence (any localizations)^9,12^.^.^ Mineralization or calcification of the globus pallidus, suspected cavernoma with popcorn pattern, or petechial hemorrhage within the infarct were not considered microbleeds^17^. WMHs were divided into periventricular and deep white matter according to Fazekas 0–3^18^, and we used the highest score as an overall measure of the severity of WMH. Perivascular spaces were assessed as fluid-filled spaces that were visible as either linear or round/ovoid CSF–like signals with an axial diameter < 3 mm that follow the orientation of penetrating arterioles in basal ganglia and centrum semiovale^12^. The severity of EPVS was assessed at the level of basal ganglia as follows: 0: no EPVS, 1: < 10 EPVS, 2: 11–20, 3: 21–40, and 4: > 40^19^. The procedure was based only on the hemisphere with more visible pathology to account for a possible asymmetry of perivascular spaces or a masking effect of the acute ischemic infarct. Usually, perivascular spaces are evaluated on the T2-sequence, which was not a standard sequence in the applied acute stroke MRI protocol, therefore we used the B0-image and ADC-map of the DWI in combination with FLAIR and T1 to count the number of perivascular spaces. We aimed to counter possible noise introduced by this limitation with the less fine-grain ranking EPVS scale (0–4) used in the SVD score^9,12^.

A binary evaluation of each SVD feature was created according to the suggested rating scheme for the total SVD burden^8,9^: the SVD features were scored 1 with the cutoffs > 0 for lacunes, > 0 for microbleeds, and ≥ 3 for EPVS rating. The binary evaluation of WMH was scored 1 with periventricular WMH = 3 or deep WMH ≥ 2^8,9^. The total SVD burden score^8,9^ was computed by adding the binary scores of the four features, creating a scale with integer values ranging from 0 to 4.

### Statistical analysis

We tested the data distribution of quantitative SVD measures with Shapiro–Wilk tests and found a significant deviation from the normal distribution for all scores. Therefore, we assessed Kendall’s nonparametric τ intercorrelation between the SVD parameters and age. For the regression analysis, we log-transformed (with ln(x) or ln(x + 1)) the quantitative measures. All non-binary regressors were z-standardized to ensure comparability in the interpretation of coefficients. The target variable stroke outcome was measured using the mRS 3 months post-stroke. As this variable is ordinal (range 0–6), we calculated ordered logistic regression with the baseline regressors age, pre-stroke functional disability (pre-stroke mRS), stroke severity 24 h after symptoms onset (NIHSS 24 h), and added SVD features. We did not introduce the application of the acute stroke treatment (= thrombolysis yes/no) and its success (TICI-grade) in the baseline model, as they are reflected in the stroke severity 24 h after symptoms onset (NIHSS 24 h) i.e. after application of the acute stroke treatment. The SVD features were assessed (i) continuously = quantitatively, or (ii) binary = qualitatively. (i) In model 1, we assessed the *continuous* or quantitative SVD measures: the transformed number of lacunes and microbleeds, and the transformed ratings for WMH (Fazekas) and EPVS respectively; (ii) in model 2, we included the four SVD features as *binary* variables. To investigate the independent impact of SVD features on stroke outcome, we used backward stepwise elimination of regressors guided by *p*-values (*p* < 0.05) to find out which SVD feature is associated with stroke outcome *independent* from other SVD lesions and clinical parameters. Additionally, we performed a principal component analysis to exclude the multicollinearity constraining the interpretation of regression analysis.

In an additional logistic regression analysis, we evaluated the predictive value of SVD scales in relation to binary long-term prognosis (favorable versus poor) to evaluate their use in bedside prognosis. We modeled the binarised mRS 3 months post-stroke, split into favorable (0–2) and poor (3–6) outcome. The area under curve (AUC) in this logistic regression and its confidence intervals allowed us to directly compare the value of full, non-hierarchical models for quantitative versus binary SVD scales versus the total SVD burden score. For direct comparability of quantitative versus binary SVD scales, we ensured that both models included the same amount of regressors. We chose models with all regressors, i.e. age, pre-stroke mRS, NIHSS 24 h, and the four SVD measures, thereby creating the most optimistic estimate for a predictive gain by SVD measures. For this analysis, we excluded 50 patients with a medium or higher degree of disability already before stroke (i.e. pre-stroke mRS > 2). The predictive value was assessed by a receiver operating characteristic on the outcome of the logistic regression. The AUC and its confidence intervals were bootstrapped with 10.000 resamplings. The likelihood ratio test was applied to compare the baseline models with those with added SVD parameters. The statistical analysis was performed in R-statistics using the MASS package^20^.

## Results

We included 873 patients in the present study. Table 1 summarizes the patients’ clinical characteristics. The pronouncement of all SVD features correlated weakly with each other (Kendall-tau, 0.283 > τ > 0.091, all *p* ≤ 0.001) and with age (Kendall-tau, 0.245 > τ > 0.109, all *p* < 0.001) except for WMH that strongly correlated with age (Kendall-tau, τ = 0.423, *p* < 0.001). An additional principal component analysis (the Jolliffe criterion, eigenvalues > 0.7) suggested that SVD features in the dataset were indeed four-dimensional enabling their further regression analysis.

**Table 1:** Patients characteristics.

Clinical and radiological variables	All participants, N = 873
Age, years mean (SD)	67.9 (15.4)
Sex, Male %	56.0
Thrombolysis, no/i.v./i.v.&MT/ MT, %	20.5/29/22/28.5
History of TIA, %	4.6
Hypertension, %	67.2
Diabetes, %	15.3
Smoking, %	27.1
Hyperlipidemia, %	69.3
Atrial Fibrillation, %	25.7
Coronary Heart Disease, %	15.3
LVO, yes %	65.4%
Body Mass Index, mean (SD)	27.1 (15.3)
Pre-stroke mRS, median (IQR; range)	0 (0, 0; 0–5)
3 months mRS, median (IQR; range)	1 (0, 2; 0–5)
NIHSS at admission, median (IQR; range)	6 (2, 11; 0–36)
NIHSS 24 h, median (IQR; range)	2 (1, 6; 0–36)
Continuous SVD assessment	
Number of lacunes, mean (SD; range)	0.46 (1.15; 0–10)
Number of microbleeds, mean (SD; range)	0.77 (3.20; 0–59)
Enlarged perivascular spaces, rating, mean (SD; range)	2.09 (0.88; 0–4)
WMH Fazekas scale, mean (SD; range)	1.28 (0.91; 0–3)
Binary SVD assessment, presence of pathology	
Lacunes, %	22.6
Microbleeds, %	23.4
Enlarged perivascular spaces^1^, %	30.2
WMH Fazekas scale^1^, %	33.2

IQR—interquartile range; NIHSS—National Institutes of Health Stoke Scale; LVO—large vessel occlusion; MT—mechanical thrombectomy; SD—standard deviation; TIA—transient ischemic attack; WMH—white matter hyperintensities.

^1^—presence of pathology according to the rating scheme for the total SVD score^8,9^.

### Independent impact of SVD features on stroke outcome

We analyzed the impact of SVD features on stroke outcome (mRS, 0–6) independent from other SVD features and baseline clinical characteristics (age, pre-stroke disability and NIHSS 24 h). The analysis of model 1 with the *continuous* assessment of SVD features demonstrated that the number of microbleeds at the time of stroke improved prediction of stroke outcome (*p* = 0.002), whereas other SVD parameters—EPVS, WMH, and the number of lacunes—did not (backward elimination, ordered logistic regression, total McFadden’s pseudo-R^2^ = 0.122, AIC = 2454.7, Table 2; Supplementary Table S1). In model 2 with the *binary* evaluation of SVD features, prediction of stroke outcome was improved by the presence of lacunes (*p* = 0.01, pseudo R^2^ = 0.121, AIC = 2457.3, Table 2; Supplementary Table S2). The models were qualitatively comparable in the deviance explained. Importantly, age, stroke severity 24 h, and pre-stroke disability demonstrated a strong impact in both models (Table 2). Post-hoc, we additionally tested a model that included both the binary SVD score for lacunes and the number of microbleeds assessed continuously together with the baseline clinical parameters age, pre-stroke disability, and stroke severity 24 h. Both the presence of lacunes (*p* = 0.03) and number of microbleeds (*p* = 0.007) significantly contributed to the model together with the baseline variables (Table 2, pseudo R^2^ = 0.123, AIC = 2452.1). The direction of effects was always in the expected direction, meaning that the presence/severity of SVD features were associated with worse stroke outcome.

**Table 2:** Results of the ordered regression models evaluating the independent impact of individual SVD features on stroke outcome.

Regressor	Odds Ratio	95% CI
**Continuous assessment of SVD features**		
No. Microbleeds	1.21	1.07–1.37
Age	1.19	1.04–1.36
NIHSS 24 h	3.24	2.79–3.77
Pre-stroke mRS	1.37	1.20–1.58
**Binary assessment of SVD features**		
Presence of lacunes (> 0)	1.48	1.10–1.99
Age	1.20	1.06–1.37
NIHSS 24 h	3.25	2.80–3.79
Pre-stroke mRS	1.36	1.19–1.56
**Post hoc analysis**		
No. Microbleeds	1.19	1.05–1.34
Presence of lacunes (> 0)	1.39	1.03–1.88
Age	1.18	1.03–1.34
NIHSS 24 h	3.27	2.82–3.81
Pre-stroke mRS	1.36	1.19–1.56

Odds ratios and lower and upper boundary of 95% confidence intervals (CI) for the normalized regressors that remained in the models after stepwise backward elimination are presented. Odds ratios refer to a change of 1 standard deviation. For the binary assessment model, the odds ratios for the presence of the corresponding pathology (e.g. at least 1 lacune present) are shown. Details on the step-wise backward regression procedure and excluded variables are reported in the Supplementary Tables.

### SVD measures in the prediction of favorable versus poor stroke outcome

**Figure 1:**
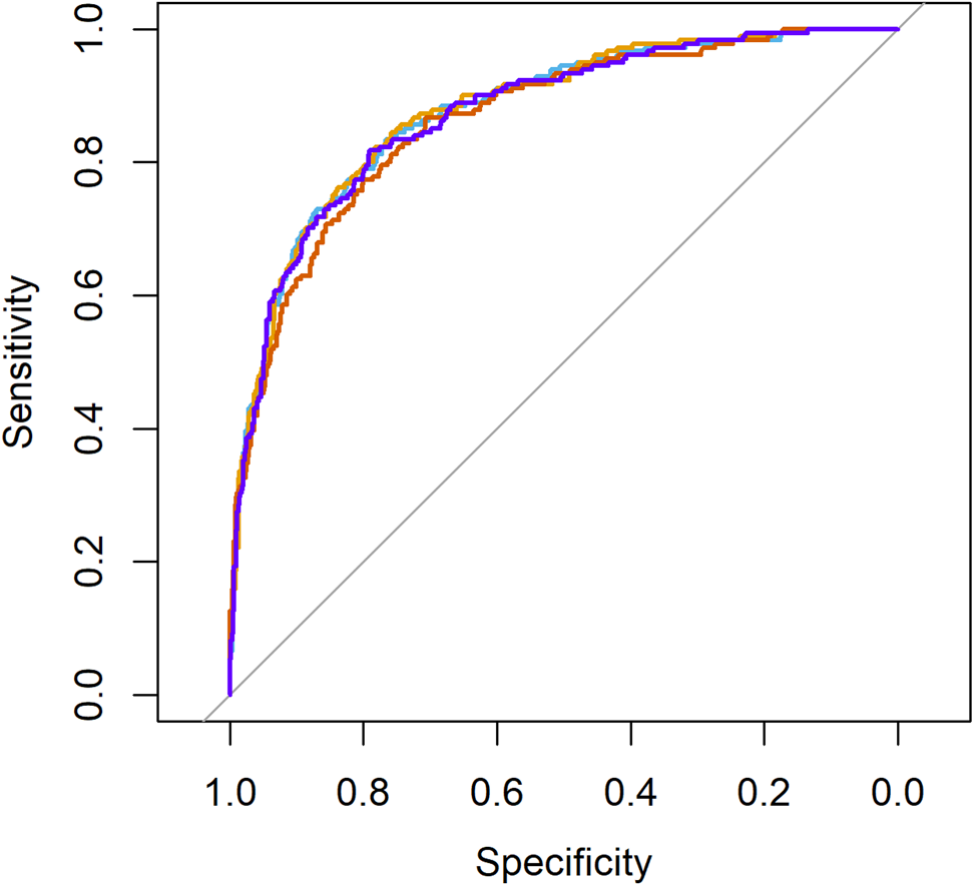
Receiver operating characteristics. Receiver operating characteristic curves that underlie the computation of the area under curve (AUC) for the logistic regression models of favorable (3-month mRS 0–2) versus poor (3-month mRS 3–6) stroke outcome. Blue: model including continuous SVD measures; yellow: model including binary rating of SVD measures; purple: model including the total SVD burden score; red: baseline model with baseline clinical parameters without any SVD measure.

We analyzed the impact of continuous and binary SVD measures in the prediction of *binary* stroke outcome, i.e. favorable (mRS 3 months 0–2) versus poor (mRS 3 months > 2) outcome. We excluded patients with pre-stroke disability (pre-stroke mRS > 2) from this analysis as they could bias the results. The comparison of AUCs between models (Fig. 1) with all continuous SVD measures (AUC = 0.876, 95% CI 0.849–0.906, pseudo R^2^ = 0.347), all binary SVD measures (AUC = 0.878, 95% CI 0.851–0.908, pseudo R^2^ = 0.348), the single total SVD burden score (AUC = 0.873, 95% CI 0.834–0.894, pseudo R^2^ = 0.311), and a baseline model without any SVD measures (AUC = 0.863, 95% CI 0.833–0.894, pseudo R^2^ = 0.325) found strongly overlapping confidence intervals between all three models that included SVD variables. Likelihood ratio tests indicated that the baseline model was outperformed by all those models—the model with continuous SVD predictors (*X*^2^(4) = 18.6, *p* < 0.001), the model with binary predictors (*X*^2^(4) = 20.0, *p* < 0.001), and the model with the total SVD burden score (*X*^2^(1) = 12.0, *p* < 0.001), though the overall model improvement was numerically small.

## Discussion

In contrast to the previous studies, the present study investigated the association of individual SVD features with functional stroke outcome adjusting for the presence and severity of other SVD markers, besides basic clinical parameters. We compared the continuous versus binary assessment of SVD features reflecting correspondingly the pronouncement and the simple presence of specific SVD features, while also taking relevant clinical parameters into account. As significant predictors of stroke outcome remained the continuous assessment of the number of microbleeds and, in the binary model, the presence of lacunes. A post hoc analysis showed that both features independently contributed to the prediction of stroke outcome when assessed with mRS 0–6. A statistically significant improvement in the simple binary prediction of stroke outcome (favorable versus poor) was observed by adding information on SVD features either only binary, only continuous, or as a total burden score, though the improvement in overall model fit was small (Fig. 1).

The number of microbleeds improved the prediction of stroke outcome, whereas the simple presence or absence of one microbleed (binary model) was not informative for the prediction model. One might explain it by the high prevalence (up to 38.3%) of microbleeds in the elderly^21^ pointing that the presence of one single microbleed could not represent a cerebrovascular pathology and therefore does not impact stroke outcome. In contrast, the number of microbleeds appears to represent a better measure of cerebrovascular pathology. The number of microbleeds showed the highest variance across assessed SVD features, and this variance was best captured by continuous measurement. This suggests that microbleeds require another cut-off in the SVD score or should be captured gradually to reflect best the SVD severity and its potential relevance on stroke outcome. For the lacunes, its presence assessed in the binary model might indicate more severe SVD^1^, whereas the number of lacunes in the continuous model was outperformed by the number of microbleeds. Another possible explanation for the low predictive value of the number of lacunes on stroke outcome might be their heterogeneity: the higher number of lacunes in the absence of microbleeds was not associated with worse stroke outcome, whereas fewer lacunes in the presence of microbleeds did^22^.

The impact of microbleeds on stroke is mainly investigated in the context of intracerebral hemorrhage. Microbleeds were associated with high mortality and morbidity^7^, an increased risk of intracerebral hemorrhage, ischemic stroke, death^4,23^, a higher risk of vascular dementia^24^, and stroke outcome^15^. As the continuous measure of microbleeds outperformed other SVD features, this parameter might be a marker for a far-progressed SVD stage.

The functional impact of lacunes was only investigated in a few studies^7^. Lacunes assessed in CT scans were found to be associated with poor stroke outcome^25^. In contrast, in a meta-analysis of patients who underwent mechanical thrombectomy for acute ischemic stroke, lacunes were not associated with poor outcome at 90 days^26^. Multiple lacunes were associated with worse outcome than single ones^14^. But again, most studies examined the impact of lacunes without taking into account other SVD features that made further implications on its independent impact difficult. In the present study, the presence of lacunes was associated with worse stroke outcome independent of age, stroke severity, pre-stroke disability and other SVD-features.

We did not observe an independent impact of WMH on stroke outcome. Historically, WMH were the first described feature of SVD^18,27^, as it could be visualized with conventional MRI sequences and CT. Therefore, WMH is the most investigated SVD feature. It was related to the risk of stroke and mortality^4^, and stroke outcome^13^. On the other hand, WMH is one of the most common incidental findings on MRI and strongly correlates with age^7^. The progression of WMH is also strongly associated with an increase of the number of lacunes^28^, and a higher burden of microbleeds^29^. Although WMH alone are associated with stroke outcome, the typically co-occurring lacunes and microbleeds outperformed the predictive value of WMH in the present regression analysis and thus could better explain clinical variance in stroke outcome. On the other hand, the volumetric analysis of WMH might further improve its predictive value and should be investigated in future research.

Although perivascular spaces are one of the recognized features of SVD, the data on its clinical relevance is still controversial^12,30^. The heterogeneous anatomy and physiology of perivascular spaces may explain the controversies^31^. The number of visible perivascular spaces on MRI increases with age, vascular risk factors (particularly hypertension), and other features of SVD^31^ that may be confounding factors complicating the assessment of the independent impact of EPVS on stroke outcome. In addition, a meta-analysis of 94 eligible studies in SVD reported limited data on EPVS, suggesting the need for further investigations^4^. In the present study, EPVS had no independent effect on stroke outcome.

The independent impact of single SVD features on stroke outcome was not investigated yet: The majority of studies investigated either the impact of one single SVD feature or the impact of total SVD burden measured via SVD sum score^9^. Several studies demonstrated the association between a higher SVD sum score with poorer stroke outcome^32^, whereas other studies failed to confirm this association for both ischemic^10,11^ and hemorrhagic stroke^33^, when compared to the usual clinical predictors, such as age and initial stroke severity (baseline NIHSS). Only a few studies assessed the impact of SVD features continuously, and the results were contradicting: one study failed to confirm this association^34^, whereas another one did^10^. In the present study, the prediction of stroke outcome (favorable versus poor) was statistically improved by adding all SVD features assessed either continuously or binarily. However, the effect of this improvement was negligible (Fig. 1). In other words, the severity of SVD lesions does not contribute substantially to poor outcome in a bedside setting but should be considered in big data studies or large trials. The small effect of adding SVD parameters might be explained by the outcome measure (favorable versus poor), which is mainly influenced by age and stroke severity. Besides differences in the sample size, the contradictory findings on SVD and stroke outcome might be explained by the applied statistical models, applied adjustment for the basic clinical parameters and pre-stroke disability. Furthermore, the cutoffs for binarization of individual SVD features introduced in the total SVD score have been chosen arbitrarily in a descriptive manner and have not been validated. Thus, the predictive value of the total SVD score might be improved by the application of improved and validated cutoffs for the individual SVD features. The current study has several strengths: first, microbleeds were assessed with SWI scans that are more sensitive than T2* MRI sequence. Second, we analyzed a relatively large cohort with standardized MRI sequences in the stroke protocol. Third, the study subjects represented the homogenous stroke population concerning stroke anatomy (anterior circulation), absence of the previous stroke, and absence of critical events within the observation interval that might worsen stroke outcome and in this way hamper data interpretation. The applied inclusion and exclusion criteria thus led to the homogeneity of the studied population and reduced the potential “composition bias” and consequently the Type II error^35^. Finally, we controlled for the pre-stroke functional disability that might be reduced due to accelerated aging, multimorbidity etc. However, the study also bears some limitations. As a standard procedure, perivascular spaces are evaluated on the T2 scans, which was not a standard MRI sequence in our acute stroke protocol. Therefore, we used the B0-image and ADC-map of the DWI scans in combination with FLAIR and T1 scans to rate the presence of EPVS. On the other hand, we assess the introduced noise as insignificant taking into account the rating procedure and strongly negative results. The anatomical distribution of microbleeds (basal ganglia versus lobar or cortical) and lacunes^36^ was not taken into consideration, as it is not required in the assessment of the SVD score; microbleeds localization was also not the aim of the present study. As we focused on hypertensive SVD (type 1) score, superficial siderosis was not taken into consideration and the criteria for cerebral amyloid angiopathy were not checked explicitly. This constrains the etiological interpretation of microbleeds. On the other hand, the cerebral amyloid angiopathy is much rarer than hypertensive SVD and its impact in the present result is unlikely to be decisive. MRI availability might introduce bias by exclusion of patients with more severe stroke or multimorbidity (= higher frailty) associated with contraindications for MRI examination or constraining its feasibility. However, these patients usually have a poor stroke outcome independent of SVD, whereas there is need to improve prediction of stroke outcome in patients with an uncertain prognosis. In the present study, the overall predictive values of models assessing mRS in the ordinal way were not high. This highlights the challenges of prediction of functional stroke outcome assessed in the ordinal form compared to the dichotomous one. On the other hand, the models included only basic clinical parameters. The analysis of lesion location or even simple lesion size^37^, vascular risk factors, and parameters of brain reserve and cognitive reserve^38^ might potentially improve model’s performance.

To conclude, whereas the reasonable theoretical framework for the assessment of SVD feature to predict stroke outcome is missing, the present study demonstrated that the method of assessment (continuous versus binary) is important and might differ across SVD features. From the major SVD features, only the number of microbleeds and the presence of lacunes were associated with stroke outcome *independent* of age, initial stroke severity, pre-stroke disability and other SVD features. Whereas the presence of lacunes is adequately assessed in the SVD sum score, microbleeds seem to require another cut-off or should be captured gradually, when better prediction of stroke outcome is needed. Simple binary prediction of stroke outcome (favorable versus poor) was improved by adding either continuous or binary SVD measures to clinical parameters. While this prediction improvement was not substantial for bedside settings, it should be considered in big data studies or large trials. Though the results should be confirmed in future studies, the present findings demonstrated the different value of the presence and severity of distinct SVD features that provide deeper insight into its pathophysiological and clinical impact on stroke outcome.

### Supplementary Information

Supplementary Tables.

## Data Availability

Upon contacting the corresponding author, the authors will share the anonymized data that support the findings of this study with qualified investigators whose proposal of data use has been approved by the independent local review committee.

## Abbreviations

mRS: Modified Rankin scale
NIHSS: National Institutes of Health Stroke Scale
SVD: Small vessel disease
WMH: White matter hyperintensities
EPVS: Enlarged perivascular spaces
