# Supplementary material for: The analysis of association between single features of small vessel disease and stroke outcome shows the independent impact of the number of microbleeds and presence of lacunes
Source: Sci Rep. 2024 Feb 10;14:3402. doi: 10.1038/s41598-024-53500-7 (PMC10858245; doi:10.1038/s41598-024-53500-7)
Supplement: Supplementary file 1 — Supplementary Tables. [file 41598_2024_53500_MOESM1_ESM.docx]

**Supplemental Table S1. Details on step-wise regression with continuous SVD features**

Summary of the step-wise regression procedure reporting the step-wise exclusion of variables including statistics. The excluded variable in each step is shown in bold. EPVS – enlarged perivascular spaces; WMH – white matter hyperintensities.

| Variables | 1^st^ step | 2^nd^ step | 3^rd^ step | 4^th^ step |
| --- | --- | --- | --- | --- |
| Lacunes | β=0.11; t=1.66; p=0.097 | β=0.11; t=1.63; p=0.103 | **β=0.12; t=1.79; p=0.073** |  |
| Microbleeds | β=0.16; t=2.50; p=0.012 | β=0.16; t=2.45; p=0.014 | β=0.17; t=2.62; p=0.008 | β=0.19; t=3.06; p=0.002 |
| EPVS | **β=-0.07; t=1.03; p=0.303** |  |  |  |
| WMH | β=0.10; t=1.31; p=0.191 | **β=0.09; t=1.16; p=0.245** |  |  |
| Age | β=0.15; t=1.86; p=0.062 | β=0.13; t=1.67; p=0.094 | β=0.17; t=2.53; p=0.011 | β=0.18; t=2.63; p=0.008 |
| Pre-stroke mRS | β=0.31; t=4.37; p<0.001 | β=0.31; t=4.41; p<0.001 | β=0.31; t=4.44; p<0.001 | β=0.32; t=4.61; p<0.001 |
| NIHSS 24h | β=1.18; t=15.34; p<0.001 | β=1.18; t=15.37; p<0.001 | β=1.18; t=15.41; p<0.001 | β=1.17; t=15.36; p<0.001 |

**Supplemental Table S2. Details on step-wise regression with binary SVD features**

Summary of the step-wise regression procedure reporting the step-wise exclusion of variables including statistics. The excluded variable in each step is shown in bold. EPVS – enlarged perivascular spaces; WMH – white matter hyperintensities.

| Variables | 1^st^ step | 2^nd^ step | 3^rd^ step | 4^th^ step |
| --- | --- | --- | --- | --- |
| Lacunes (binary) | β=0.35; t=2.29; p=0.022 | β=0.35; t=2.30; p=0.022 | β=0.36; t=2.38; p=0.017 | β=0.39; t=2.57; p=0.010 |
| Microbleeds (binary) | β=0.22; t=1.41; p=0.158 | β=0.21; t=1.38; p=0.168 | **β=0.23; t=1.55; p=0.122** |  |
| EPVS (binary) | **β=-0.08; t=0.59; p=0.555** |  |  |  |
| WMH (binary) | β=0.21; t=1.37; p=0.170 | **β=0.20; t=1.31; p=0.191** |  |  |
| Age | β=0.14; t=1.86; p=0.063 | β=0.13; t=1.78; p=0.075 | β=0.17; t=2.52; p=0.012 | β=0.18; t=2.57; p=0.006 |
| Pre-stroke mRS | β=0.31; t=4.42; p<0.001 | β=0.31; t=4.43; p<0.001 | β=0.31; t=4.44; p<0.001 | β=0.31; t=4.45; p<0.001 |
| NIHSS | β=1.18; t=15.38; p<0.001 | β=1.18; t=15.39; p<0.001 | β=1.18; t=15.37; p<0.001 | β=1.18; t=15.36; p<0.001 |
